# Meta-Analysis of VTE Risk: Ovarian Cancer Patients by Stage, Histology, Cytoreduction, and Ascites at Diagnosis

**DOI:** 10.1155/2020/2374716

**Published:** 2020-09-03

**Authors:** Kristin S. Weeks, Emma Herbach, Megan McDonald, Mary Charlton, Marin L. Schweizer

**Affiliations:** ^1^Department of Epidemiology, College of Public Health, University of Iowa, Iowa City 52242, IA, USA; ^2^Carver College of Medicine, University of Iowa, Iowa City 52242, IA, USA; ^3^Department of Obstetrics and Gynecology, University of Iowa Hospitals and Clinics, Iowa City 52242, IA, USA; ^4^Center for Access and Delivery Research and Evaluation, Iowa City VA Health Care System, Iowa City 52242, IA, USA

## Abstract

Venous thromboembolisms (VTEs) have been a leading secondary cause of death among ovarian cancer patients, prompting multiple studies of risk factors. The objective of this meta-analysis is to quantify the associations between VTE and the most commonly reported risk factors among ovarian cancer patients. PubMed, Embase, and Cumulative Index to Nursing and Allied Health Literature (CINAHL) were used to identify observational studies. Two reviewers independently abstracted data and assessed quality via the Newcastle–Ottawa tool. A random effects model was used to calculate the pooled odds ratios for VTE with each of the following exposures: advanced cancer stage, clear cell histology, serous histology, ascites at diagnosis, and complete cytoreduction. The *I*^2^ and *Q* tests were used to evaluate heterogeneity. Twenty cohort studies with 6,324 total ovarian cancer patients, 769 of whom experienced a VTE, were included. The odds of VTE in ovarian cancer patients were higher among patients with cancer stage III/IV (versus cancer stage I/II, pooled odds ratio (OR) 2.73; 95% CI 1.84–4.06; *I*^2^= 64%), clear cell (versus nonclear cell) histology (OR 2.11; 95% CI 1.55–2.89; *I*^2^ = 6%), and ascites (versus no ascites) at diagnosis (OR 2.12; 95% CI 1.51–2.96; *I*^2^ = 32%). Serous (versus nonserous) histology (OR 1.26; 95% CI 0.91–1.75; *I*^2^ = 42%) and complete (versus incomplete) cytoreduction (OR 1.05; 95% CI 0.27–4.11; *I*^2^ = 88%) were not associated with VTE. This meta-analysis quantifies the significantly elevated odds of VTE in ovarian cancer patients with advanced stage at diagnosis, clear cell histology, and ascites at diagnosis. Further studies are needed to account for confounders and inform clinical decision-making tools.

## 1. Introduction

Venous thromboembolisms (VTEs), including deep vein thromboses (DVTs) and pulmonary embolisms (PEs), are a major complication of the hypercoagulative state caused by cancer [1, 2]. Ovarian cancer patients have one of the highest rates of VTEs among all cancer patients [3–10]. Additionally, VTEs are a leading secondary cause of death for ovarian cancer patients and can cause significant morbidity and decreased quality of life [9, 11–17].

There are many reasons ovarian cancer patients are at high risk for VTEs [8]. First, eighty percent of women with ovarian cancer are diagnosed at an advanced cancer stage with regional or distant metastasis [18]. Large tumor growth and accumulated ascites at diagnosis can compress the pelvic veins in women leading to hemostasis and increased risk of thrombosis [19, 20]. Moreover, advanced stage at diagnosis and the associated abdominal ascites are thought to contribute to VTE events through cellular mechanisms, vessel wall irritation, inflammation, and thrombocytosis [8, 19, 21]. Secondly, certain ovarian cancer histologies, such as clear cell carcinoma, are also thought to contribute to hypercoagulability and endothelial risk [19, 22]. The grade, aggressiveness, and cellular-level mechanisms associated with specific histological types of ovarian cancer are thought to influence VTE risk through the upregulation of tissue factor, VIIa, biomarkers, and macrophages [9, 15, 18, 19, 23, 24]. Further, the aggressive surgical and chemotherapy treatment of ovarian cancer likely causes high thrombosis rates [16, 19, 25–27]. Complete cytoreductive surgery, the recommended surgical treatment for ovarian cancer, requires extensive surgical steps involving lymph node sampling, tumor removal, and organ removal [28, 29].

Estimates of the odds of VTE in ovarian cancer patients by common tumor, clinical presentation, and treatment factors have sparsely been reported over the last three decades, most often only including unadjusted odds ratios or raw numbers. The odds of VTEs in ovarian cancer patients by the most frequently discussed tumor, clinical presentation, and treatment factors need to be quantified by meta-analysis in order to further our understanding of VTE hazard in this high-risk population [30]. This meta-analysis of observational studies aims to examine the risk of VTE in ovarian cancer patients by the exposures of advanced stage at diagnosis, serous histology, clear cell carcinoma histology, ascites at diagnosis, and complete cytoreductive surgery.

## 2. Materials and Methods

### 2.1. Data Sources and Searches

Human studies published before April 17, 2019, detailing risk factors associated with VTE in ovarian cancer patients were identified from PubMed, Embase, and Cumulative Index to Nursing and Allied Health Literature (CINAHL). Reference lists were reviewed, although this only provided duplicates. The primary index terms used were “ovarian neoplasm,” “carcinoma, ovarian epithelial,” and “venous thromboembolism.” A medical librarian assisted in creating our search strategy (Appendix A). We did not restrict by date; however, the terms were created in the databases in 1974. No studies published prior to 1990 were found. Published conference abstracts were included in our search. Unpublished studies were not sought, and no authors were contacted. Studies were not restricted by language.

### 2.2. Study Selection

Studies eligible for inclusion met the following criteria: women; exclusively malignant ovarian cancer patients; multiple (>2) histologies represented; all four cancer stages represented; and pulmonary embolisms, deep vein thromboses, a combination of both, or generalized VTEs as an outcome. Only observational studies were included. Studies were excluded if they provided only aggregate results for multiple cancer types, compared ovarian cancer patients to noncancer patients, and investigated an experimental intervention beyond standard of care. Other exclusion criteria included the lack of at least one exposure of interest, the inability to calculate an odds ratio, and spontaneous arterial thrombosis.

The exposures of interest (and the comparator groups) were cancer stage III/IV (cancer stage I/II), serous histology (nonserous histology), clear cell histology (nonclear cell histology), cytoreduction with total removal of the tumor (cytoreduction without total removal of the tumor), and significant ascites of the abdomen at diagnosis (unremarkable ascites of the abdomen). Ascites was defined as any volume measurement cutoff or by the clinical judgement of the physicians in the study.

### 2.3. Data Abstraction

The variables in the abstraction form included authors, publication year, study location, study design, the number of healthcare centers included, interventions received, anticoagulation, how VTE was diagnosed (whether by presentation with symptoms or screening), time period of study, VTE type, timing of the VTE in the treatment/diagnosis course, the average age of those with and without the outcome, frequencies and/or effect estimates (unadjusted and adjusted), and two-by-two contingency tables for each exposure and the outcome. Abstraction forms were piloted by one reviewer ahead of abstraction.

One investigator determined eligibility of studies. Two investigators independently extracted the data. Each investigator was blinded to the other investigator's codes. A third investigator reviewed the differences and recorded the number of disagreements. The abstractors reviewed and revised their responses independently. All the remaining disagreements were reconciled by consensus. Each study's quality was evaluated using the Newcastle-Ottawa risk of bias assessment tool for cohort studies.

### 2.4. Statistical Analysis

Unadjusted odds ratios and raw numbers were primarily reported in articles; thus, this study pooled unadjusted odds ratios. Pooled odds ratios for VTE in ovarian cancer patients by each exposure were computed using random effects models with inverse-variance weighting. The *I*^2^ and Cochrane *Q* tests were used to assess heterogeneity. The potential for publication bias was evaluated using funnel plots. Clear cell carcinoma was the only exposure that had more than one reported multivariable odds ratio. We completed a sensitivity analysis in which we pooled adjusted odds ratio results for clear cell carcinoma (versus other histologies).

Results were stratified by those that diagnosed VTE at least partially by screening asymptomatic patients versus diagnosed only symptomatic patients. If three or more articles within each stratum assessed an exposure, a stratified analysis was conducted. If less than three articles were in each stratum, we conducted a subgroup analysis; this was completed for the serous histology exposure. This stratification scheme was chosen *a priori* for clinical relevance.

## 3. Results

### 3.1. Literature Search and Study Characteristics

**Figure 1:**
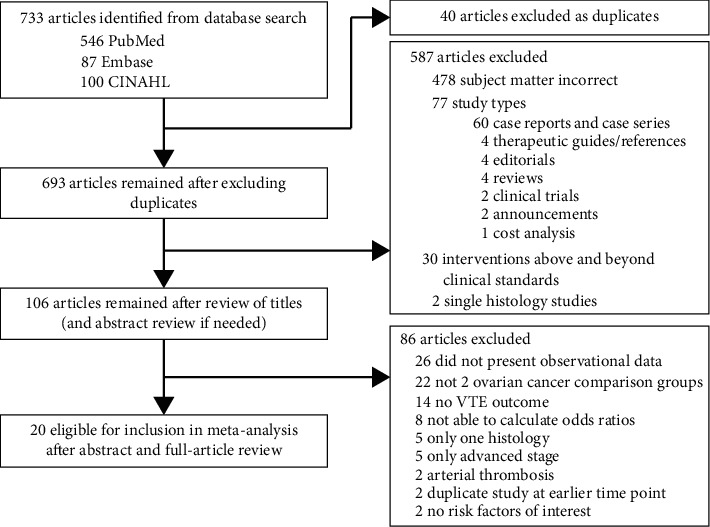
Selection of studies in the meta-analysis. This figure shows the selection criteria and process. 733 articles were identified and ultimately 20 articles were included in the study.

There were 733 articles identified in the database search; 106 full-text articles were reviewed, and 20 articles met eligibility criteria (Figure 1) [13–15, 23, 26, 31–47]. The included studies were performed in the following countries: Japan (*N* = 7), the United States (6), China (2), Italy (2), England (1), Ireland (1), and Germany (1) (Table 1). The majority (16) were single-institution, retrospective cohort studies of moderate quality (Table 2). The earliest period when a patient was diagnosed with ovarian cancer in any study was 1990 and the latest was 2017. There was variability in the proportion of each study population that received anticoagulation, as well as the length, type, and timing of anticoagulation used. Treatment received, method of VTE identification, and the timing of VTE events in relation to treatment and/or diagnosis also varied among studies. The retrospective studies primarily assessed VTE after surgical treatment with or without chemotherapy concurrence. The four prospective studies assessed presurgery VTE events (Table 1).

There were 6,324 ovarian cancer patients included in the main analysis and 769 patients experienced a VTE. It was not possible to calculate an average age across studies because it was not always reported, but most studies estimated a mean age of around 60 years of age. There were three separate instances of overlapping study populations (Sakurai and Satoh, Tateo and Mereu, and Wagner and Mokri). If the overlapping studies evaluated one of the same exposures, the study with the larger sample size was used in that exposure analysis. The smaller study was used when the larger overlapping study did not assess an exposure.

### 3.2. Meta-Analysis Results

**Figure 2:**
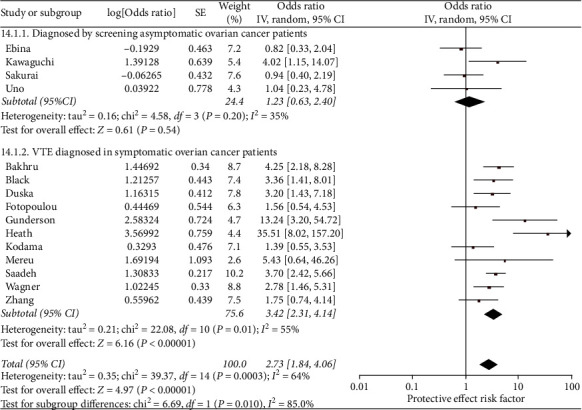
Forest plot of the odds of VTE in ovarian cancer patients that have cancer of stages III and IV at diagnosis (versus stages I and II). This figure shows the pooled odd of stage III/IV cancer (versus stage I/II). The total pooled odds are 2.73 (1.84-4.06).

Ovarian cancer patients with cancer stage III/IV at diagnosis had 2.73 times greater pooled odds of having a VTE than patients with ovarian cancer stage I/II at diagnosis (95% confidence interval (CI) 1.84–4.06, number of studies (*N*) = 15) (Figure 2). Overall, the exposure had moderate-to-high heterogeneity (*I*^2^ = 64%). Stratified analyses were significantly different (*p* = 0.01). The publications that diagnosed VTE by screening asymptomatic patients had lower heterogeneity (*I*^2^ = 35% vs. 55%) than the symptomatic subgroup and a nonsignificant pooled odds ratio (OR 1.23, 95% CI 0.63–2.40, *N* = 4). The stratum of articles with symptomatic patients had a significantly elevated pooled odds ratio (OR 3.42, 95% CI 2.31–5.06, *N* = 11).

**Figure 3:**
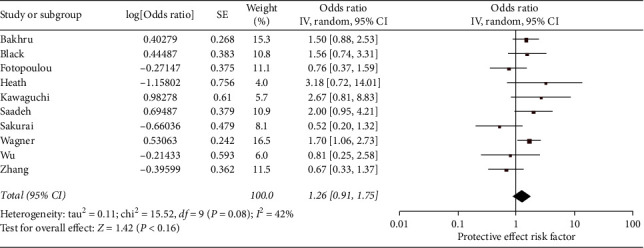
Forest plot of the odds of VTE in ovarian cancer patients that have serous histology tumors (versus nonserous histologies). This figure shows the pooled odd of serous histology (versus nonserous histology). The total pooled odds are 1.26 (0.91–1.75).

The overall pooled odds of VTE in ovarian cancer patients with a serous histology were nonsignificant at 1.26 times greater odds of VTE than other histologies (95% CI 0.91–1.75, *N* = 10) (Figure 3). Heterogeneity overall was moderate (*I*^2^ = 42%) for serous histology. The subgroup analysis (*N* = 8, removed Kawaguchi and Sakurai) of articles with VTE diagnosed in symptomatic patients was significant (OR 1.35, 95% CI 1.05–1.72). Subgroup heterogeneity was mildly reduced to *I*^2^ of 32% (not shown).

**Figure 4:**
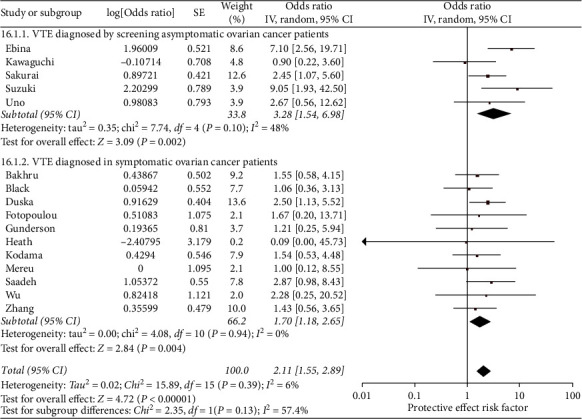
Forest plot of the odds of VTE in ovarian cancer patients that have clear cell carcinoma histology (versus nonclear cell carcinoma). This figure shows the pooled odd of clear cell carcinoma histology (versus nonclear cell carcinoma). The total pooled odds are 2.11 (1.55-2.89).

The pooled odds of VTE in ovarian cancer patients with a clear cell carcinoma histology were 2.11 (95% CI 1.55–2.89, *N* = 16) times greater than ovarian cancer patients without that histology (Figure 4). Heterogeneity overall was minimal (*I*^2^ = 6%). Odds of VTE by clear cell histology were not significantly different between patients diagnosed by screening and by symptomatic presentation (*p*=0.13), and both had odds ratios significantly greater than one. The pooled odds ratio for the articles with asymptomatic VTE diagnoses was 3.28 (95% CI 1.54–6.98), while it was 1.70 (95% CI 1.18–2.46) for the articles with symptomatic diagnoses. The clear cell carcinoma stratum with subclinical diagnosis (*N* = 5) had greater heterogeneity (*I*^2^ = 48% vs. 0%) than the symptomatic stratum (*N* = 11). The sensitivity analysis using only multivariable odds ratios found elevated risk, with clear cell carcinoma having 6.29 times greater odds of VTE than other histologies (95% CI 3.08–12.85, *N* = 4).

**Figure 5:**
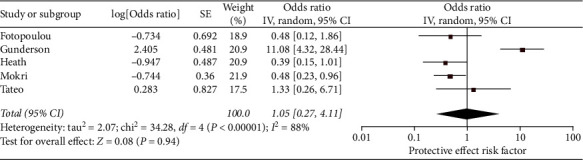
Forest plot of the odds of VTE in ovarian cancer patients that have complete cytoreduction surgery with no tumor mass left (versus incomplete cytoreduction with tumor mass left). This figure shows the pooled odd of complete cytoreduction (versus incomplete cytoreduction). The total pooled odds are 1.05 (0.27–4.11).

There was no significant difference in the odds of VTE related to whether the patient had complete tumor removal with cytoreductive surgery (OR 1.05, 95% CI 0.27–4.11, *N* = 5) versus did not have total tumor removal (Figure 5). Substantial heterogeneity was present (*I*^2^ = 88%).

**Figure 6:**
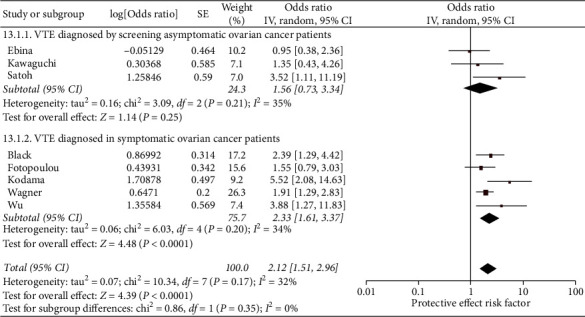
Forest plot of the odds of VTE in ovarian cancer patients that have ascites at diagnosis (versus nonsignificant ascites). This figure shows the pooled odd of ascites at diagnosis (versus nonsignificant ascites). The total pooled odds are 2.12 (1.51-2.96).

Ovarian cancer patients with ascites at diagnosis had 2.12 (95% CI 1.51–2.96, *N* = 8, *I*^2^ = 32%) times greater odds of having a VTE than patients without ascites at diagnosis (Figure 6). Within publications with subclinical VTE diagnoses, there was low-to-moderate heterogeneity and a nonsignificant pooled odds ratio (OR 1.56, 95% CI 0.73–3.34, *N* = 3, *I*^2^ = 35%). The publications with symptomatic VTEs had low-to-moderate heterogeneity and a significantly elevated pooled odds ratio (2.33, 95% CI 1.61–3.37, *N* = 5, *I*^2^ = 34%).

### 3.3. Publication Bias

All funnel plots showed mild asymmetry, meaning publication bias could not be ruled out (Supplemental Figure 1).

## 4. Discussion

The results of this meta-analysis suggest that advanced cancer stage, clear cell carcinoma histology, and ascites at diagnosis are significant risk factors for VTE events in ovarian cancer patients. The odds ratios for advanced cancer stage and ascites at diagnosis were significant overall and for publications where only symptomatic patients were diagnosed with VTE. It is possible that the results of asymptomatic patients were limited by the few studies available or represent a racial difference as these results were all from Japan. Given that the asymptomatic stratums had uneven distributions in their funnel plots, it is possible that publication bias could have contributed to the limited number of studies. Finally, these results may suggest that advanced cancer stage and ascites at diagnosis increase the odds of severe VTEs that cause symptoms, such as pain, swelling, and dyspnea versus the odds of all coagulopathy.

Histologically, clear cell carcinoma significantly increased the odds of VTE in both publications that diagnosed VTE in asymptomatic patients and symptomatic patients. This finding has been supported by cellular-level mechanism studies of genes, tissues factors, and inflammatory activation [48–52]. The moderate-to-high heterogeneity seen among the subclinical stratum may have been caused by variability in ultrasound technician skills, equipment used, patient population BMI differences, or study quality. Given the large number of studies and the absence of heterogeneity among publications, it is likely that across various countries, treatments, and patient populations, clear cell carcinoma histology increases the odds of VTE in ovarian cancer patients. Finally, our sensitivity analysis suggests that the true independent odds of VTE attributed to clear cell carcinoma are likely even greater than the pooled bivariate results.

There was no significant difference in the overall pooled odds of VTE related to serous histology. This aligns with prior literature [33, 35]. There may be no association between serous histology and VTE risk. The significant finding for symptomatic patients may be due to confounding by stage at diagnosis [53]. Serous histology has the highest likelihood of being diagnosed at a distant stage and the lowest likelihood of being diagnosed at a local stage compared to other epithelial histologies and nonepithelial histologies [53]. A multivariable analysis assessing the risk of VTE for women with serous histology cancers would be helpful.

There was no significant difference in the pooled odds of VTE associated with receipt of complete cytoreduction. There was a high degree of heterogeneity between the pooled studies. Variability in postoperative anticoagulation, lengths of surgeries, and hospitalization rates/lengths could have affected the magnitude of the effect detected. The insignificant results may be due to a lack of association between complete cytoreduction and VTE. However, they may also be due to study limitations, including a small number of studies investigating this exposure, the use of unadjusted odds ratios, large heterogeneity between studies, and likely publication bias. Future studies are needed investigating this relationship that control for treatment differences, tumor factors, and hospital care variables.

This meta-analysis includes studies of moderate quality. The strengths of the studies included in this meta-analysis were that they independently linked their outcome to exposures through medical records and included many countries (with diverse racial/ethnic compositions), years of diagnosis, and treatment methods. The major limitations of the studies included are the temporality of the exposures and outcome, limited generalizability, and failure to control for residual confounding. Only five studies screened patients to ensure they did not have VTEs prior to the period of outcome assessment and/or excluded persons with medical conditions, falls, or surgeries that would have independently caused VTEs prior to diagnosis. Moving forward, a prospective, multicenter cohort study that screens for VTEs with questionnaires or ultrasounds at time of diagnosis could be useful. The majority of the included studies were performed at a single tertiary institution or academic teaching center. These findings may not be applicable to patients in a community hospital population or a true community population. The majority of results presented by the studies in this meta-analysis are bivariate, unadjusted results. Comprehensive patient, tumor, and clinical treatment/intervention variables, including chemotherapy variables, need to be collected and analyzed independent of other exposures in a multivariable analysis.

Our results quantify the difference in pooled odds of VTE in ovarian cancer patients by the most commonly reported exposures. An estimated 5%–25% of ovarian cancer patients will have a VTE within the first two years after their cancer diagnosis [13, 17, 54, 55]. These women will likely have lower survival rates than their counterparts without VTEs [13, 17, 54, 55]. There is a critical and urgent need to investigate the clinically relevant VTE risk factors of ovarian cancer patients in order to compile a comprehensive understanding of which ovarian cancer patients are most at risk of VTEs. These findings need to be translated into clinical decision-making tools that can improve the timeliness of detection of VTEs and subsequently reduce the risk of thrombosis-related mortality in this vulnerable population. Furthermore, these findings need to be translated into anticoagulation decision-making tools for pre- and postoperative ovarian cancer patients. The most commonly used anticoagulation risk assessment tools for ovarian cancer patients are nonspecific or do not include important variables such as histology [56–62]. Most tools were created for generalized abdominal/pelvic surgery or oncology patients [56–59, 61]. Risk assessment tools specific to ovarian cancer patients that have ascites at diagnosis, stage at diagnosis, and histology of tumor may be useful [62, 63]. In the meantime, robust multidisciplinary team efforts should consider the benefits and risks of various pre- and postoperative anticoagulation regimens [19].

## 5. Conclusions

Advanced cancer stage, clear cell carcinoma, and ascites at diagnosis significantly increased the pooled odds of VTE in ovarian cancer patients. Further studies are needed to account for confounders and inform clinical decision-making tools and anticoagulation recommendations. The possibility of publication bias could not be excluded as a limitation of this meta-analysis.

## Figures and Tables

**Table 1:** Description of the included studies.

Author, year	Study type	N VTE/N total	Location of study	Timing of study	Pharmacologic anticoagulation	Timing of VTE	Outcome	Diagnosis
Bakhru, 2012	Retrospective cohort	69/641	Michigan, USA; University of Michigan Medical Center	1999–2009	Unknown	Postoperative	DVT	Symptomatic evaluation

Black, 2007	Retrospective cohort	57/559	New York, USA; Gynecologic Service Database of Memorial Sloan-Kettering Cancer Center	01/1999–04/2015	Heparin given at surgeon's discretion	Diagnosis to 30 days Postoperative	PE, DVT	Symptomatic evaluation

Duska, 2009	Retrospective cohort	37/129	Massachusetts, USA; Massachusetts General Hospital	1994–2004	All patients received pre- and postoperative subcutaneous heparin	Over the clinical course of disease until death, loss to follow-up, or 2004	PE, DVT	Symptomatic evaluation

Ebina, 2018	Prospective cohort	25/110	Kobe, Japan; Kobe University Hospital	6/2010–12/2016	Unknown	Prior to the start of any treatment	DVT, DVT, and PE	Screening with ultrasound and CT scan

Fotopoulou, 2009	Retrospective cohort	37/525	Germany; two clinical trials of the North-Eastern German Society of Gynecologic Oncology Ovarian Cancer Study Group	09/1999–08/2005	Prophylactic care to patient's physician preference	During 2^nd^ line topotecan-based chemotherapy	VTE	Symptomatic evaluation

Gunderson, 2014	Retrospective cohort	94/586	Oklahoma, USA; University of Oklahoma Health Sciences Center	01/1996–06/2011	Unknown	At diagnosis through last follow-up	PE, DVT	Symptomatic evaluation

Heath, 2015	Retrospective cohort	19/397	London, UK; Royal Marsden Hospital and St. George's Hospital	01/2006–12/2012	Unknown	Pre- and postoperative	PE, DVT	Screening and symptomatic evaluation

Kawaguchi, 2012	Retrospective cohort	14/87	Nara, Japan; Shizuokas Cancer Center Hospital	07/2007–10/2008	Unknown	preoperative and pretreatment	DVT, DVT, and PE	Screening using ultrasound and CT scan

Kodama, 2012	Retrospective cohort	23/114	Okayama, Japan; Okayama University Hospital	08/2005–08/2011	Unknown	preoperative	VTE	High D-dimer and clinically symptomatic evaluation

Mereu, 2009	Retrospective cohort	16/203	Pavia, Italy; San Matteo Hospital	1990–2004	Surgical patients received heparin for 7 days after surgery	During chemotherapy treatment	PE, DVT	Evaluated on clinical suspicion of disease

Mokri, 2013	Retrospective cohort	35/569	Minnesota, USA; Mayo Clinic Hospital	01/2003–12/2008	To surgeon's preference	Postoperative	PE, DVT	Symptomatic evaluation

Saadeh, 2013	Retrospective cohort	33/344	Dublin, Ireland; St. Jame Hospital	2006–2010	Surgical patients received heparin	Diagnosis to 8 months, variable treatments received in time frame	PE, DVT	Routine staging CT and symptomatic evaluation

Sakurai, 2017	Prospective cohort	31/128	Tsukuba, Japan; University of Tsukuba Hospital	11/2004–12/2010	Unknown	Postoperative	DVT, DVT, and PE	Screening with ultrasound, CT and MRI

Satoh, 2007	Prospective cohort	18/72	Tsukuba, Japan; University of Tsukuba Hospital	11/2004–03/2007	Unknown	Prior to treatment and surgery	DVT, PE	Screening with ultrasound, CT and MRI

Suzuki, 2010	Retrospective cohort	21/144	Kanagawa, Japan; St. Marianna University School of Medicine Department of Obstetrics and Gynecology	01/2005–06/2008	Some patients received presurgery anticoagulation	Preoperative	DVT, PE	Screening CT and ultrasound in patients with high D-dimer levels before surgery

Tateo, 2005	Retrospective cohort	42/253	Pavia, Italy; San Matteo Hospital	1990–2001	Heparin for at least 7 days after surgery	Diagnosis until follow-up; average follow-up or time even was 24.3 months	DVT, PE	Symptomatic evaluation on clinical suspicion

Uno, 2007	Prospective cohort	10/32	Tsukuba, Japan; University of Tsukuba Hospital	01/2004	Unknown	preoperative	DVT, DVT, and PE	Screening with ultrasound, CT and MRI

Wagner, 2014	Retrospective cohort	140/860	Minnesota, USA; Mayo Clinic Hospital	01/2003–12/2011	To surgeon's preference	6 months after primary debulking surgery	DVT, PE	Symptomatic evaluation; self-report; next-of-kin report; autopsy

Wu, 2013	Retrospective cohort	13/183	Shanghai, China; Fudan University Cancer Hospital	07/2007–01/2011	Unknown	Diagnosis to at least 5 months postoperative	DVT, PE	Self-report by telephone and outpatient chart review (symptomatic evaluation)

Zhang, 2018	Retrospective cohort	35/388	Shandong, China; Qilu Hospital of Shandong University	01/2014–01/2017	Preoperative VTE cases were given heparin, 7 days of heparin given after surgery	Diagnosis to last chemotherapy treatment (>6 months)	DVT, PE	Preoperative screening with ultrasound and symptomatic evaluation after surgery

This table details the studies included in this meta-analysis. This table details the risk of bias by manuscript according to the Newcastle–Ottawa cohort study assessment tool. Two stars (or points) were possible in the “on basis of design” category, whereas only one point (or star) was possible in the other categories.

**Table 2:** Risk of bias assessment tool.

Author	Selection				Comparability	Outcome		
	Representative of exposed	Selection of nonexposed	Ascertainment of exposed	Demonstration outcome was not present at start	On basis of design	Assessment	Follow-up length	Adequacy of follow-up
Bakhru	^*∗*^	^*∗*^	^*∗*^			^*∗*^	^*∗*^	
Black		^*∗*^	^*∗*^			^*∗*^	^*∗*^	
Ebina	^*∗*^	^*∗*^	^*∗*^	^*∗*^		^*∗*^	^*∗*^	^*∗*^
Duska	^*∗*^	^*∗*^	^*∗*^		^*∗∗*^	^*∗*^	^*∗*^	
Fotopoul		^*∗*^	^*∗*^			^*∗*^	^*∗*^	^*∗*^
Gunderson		^*∗*^	^*∗*^			^*∗*^	^*∗*^	
Heath		^*∗*^	^*∗*^	^*∗*^		^*∗*^	^*∗*^	
Kawaguchi		^*∗*^	^*∗*^			^*∗*^	^*∗*^	^*∗*^
Kodama		^*∗*^	^*∗*^			^*∗*^	^*∗*^	^*∗*^
Mereu		^*∗*^	^*∗*^			^*∗*^	^*∗*^	^*∗*^
Mokri		^*∗*^	^*∗*^	^*∗*^		^*∗*^		
Saadeh	^*∗*^	^*∗*^	^*∗*^	^*∗*^		^*∗*^	^*∗*^	
Sakurai		^*∗*^	^*∗*^			^*∗*^	^*∗*^	^*∗*^
Satoh	^*∗*^	^*∗*^	^*∗*^			^*∗*^	^*∗*^	^*∗*^
Suzuki		^*∗*^	^*∗*^			^*∗*^	^*∗*^	^*∗*^
Tateo		^*∗*^	^*∗*^		^*∗∗*^	^*∗*^		^*∗*^
Uno		^*∗*^	^*∗*^			^*∗*^	^*∗*^	^*∗*^
Wagner		^*∗*^	^*∗*^	^*∗*^				
Wu		^*∗*^	^*∗*^				^*∗*^	^*∗*^
Zhang		^*∗*^	^*∗*^			^*∗*^	^*∗*^	^*∗*^

^*∗*^A point was given. ^*∗∗*^Two points were given. Blank units indicate that a point was not given.

## Data Availability

Appendix A should be referenced to obtain all the underlying data supporting the results.

## Appendix

### A. Database Search Terms Used without Explosion of Mesh Terms

PubMed search: 546 results on 4/17/19
    ((((“Ovarian Neoplasms”[Mesh:noexp]) OR “Carcinoma, Ovarian Epithelial”[Mesh])) OR (“ovarian cancer” OR “ovarian cancers” OR “ovarian neoplasm” OR “ovarian neoplasms” OR “ovary neoplasm” OR “ovary neoplasms” OR “ovary cancer” OR “ovary cancers” OR “cancer of the ovary” OR “cancer of ovary”)) AND ((((“venous thrombosis” OR “venous thromboses” OR “deep vein thrombosis” OR “deep vein thromboses” OR “deep-venous thrombosis” OR “deep-venous thromboses” OR “deep venous thrombosis” OR “deep venous thromboses”)) OR (“venous thromboembolism” OR “pulmonary embolism” OR “pulmonary embolisms” OR “pulmonary thromboembolisms” OR “pulmonary thromboembolism”)) OR (((((“Venous Thromboembolism”[Mesh]) OR “Pulmonary Embolism”[Mesh:noexp]) OR “Embolism”[Mesh:noexp]) OR “Thromboembolism”[Mesh]) OR “Thrombosis”[Mesh]))
Embase search: 87 results on 4/17/19
    (((((((“ovary”/exp OR ovary) AND (“cancer”/exp OR cancer) OR cancer) AND (“ovary”/exp OR ovary) OR malignant) AND (“ovary”/exp OR ovary) AND (“tumor”/exp OR tumor) OR malignant) AND (“ovary”/exp OR ovary) AND (“tumour”/exp OR tumour) OR ovarial) AND (“cancer”/exp OR cancer) OR ovarian) AND (“cancer”/exp OR cancer) OR “ovarium”/exp OR ovarium) AND (“cancer”/exp OR cancer) AND (((((((venous AND (“thromboembolism”/exp OR thromboembolism) OR thromboembolism) AND venous OR “vein”/exp OR vein) AND (“thromboembolism”/exp OR thromboembolism) OR deep) AND (“vein”/exp OR vein) AND (“thrombosis”/exp OR thrombosis) OR lower) AND (“extremity”/exp OR extremity) AND deep AND (“vein”/exp OR vein) AND (“thrombosis”/exp OR thrombosis) OR “lung”/exp OR lung) AND (“embolism”/exp OR embolism) OR upper) AND (“extremity”/exp OR extremity) AND deep AND (“vein”/exp OR vein) AND (“thrombosis”/exp OR thrombosis) OR lower) AND (“extremity”/exp OR extremity) AND deep AND (“vein”/exp OR vein) AND (“thrombosis”/exp OR thrombosis)
CINAHL: 100 results on 4/17/19
    (venous thromboembolism OR deep vein thrombosis OR deep vein thrombosis OR deep venous thrombosis OR deep-venous thromboses OR pulmonary embolism OR pulmonary thromboembolism) AND (ovarian cancer OR ovarian cancer OR ovary cancer OR ovarian neoplasm OR carcinoma, ovarian epithelial OR cancer of the ovary OR cancer of ovary).

## Supplementary Materials

Supplementary Materials Supplemental Figure 1: funnel plots of all included exposures. This figure shows the funnel plots for all exposures. There is mild asymmetry indicating publication bias cannot be ruled out.
