# Supplementary material for: Meta-Analysis of VTE Risk: Ovarian Cancer Patients by Stage, Histology, Cytoreduction, and Ascites at Diagnosis
Source: Obstet Gynecol Int. 2020 Sep 3;2020:2374716. doi: 10.1155/2020/2374716 (PMC7486642; doi:10.1155/2020/2374716)
Supplement: Supplementary Materials — Supplemental Figure 1: funnel plots of all included exposures. This figure shows the funnel plots for all exposures. There is mild asymmetry indicating publication bias cannot be ruled out. [file 2374716.f1.pdf]

Supplemental Figure 1. Funnel Plot of all included exposures

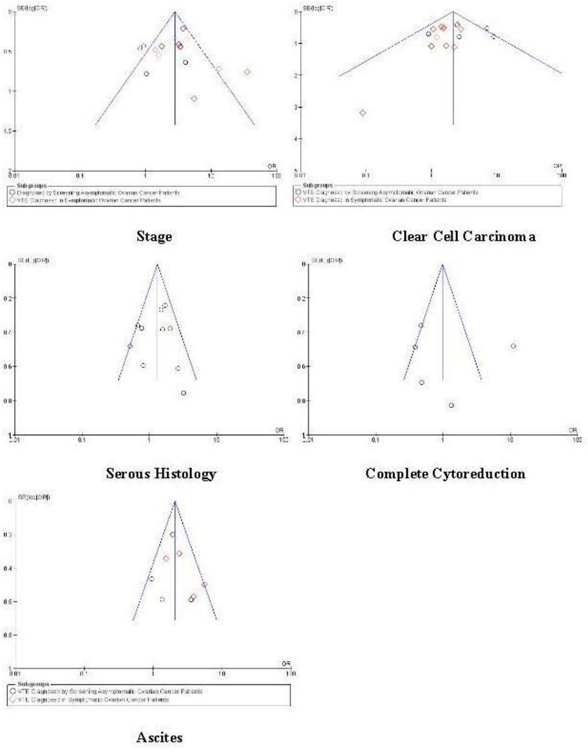


This figure shows the funnel plots for all exposures. There is mild asymmetry indicating publication bias cannot be ruled out.
